# Nicotine Enhances *Staphylococcus epidermidis* Biofilm Formation by Altering the Bacterial Autolysis, Extracellular DNA Releasing, and Polysaccharide Intercellular Adhesin Production

**DOI:** 10.3389/fmicb.2018.02575

**Published:** 2018-10-29

**Authors:** Yang Wu, Yue Ma, Tao Xu, Qing-zhao Zhang, Jinna Bai, Jiaxue Wang, Tao Zhu, Qiang Lou, Friedrich Götz, Di Qu, Chun-quan Zheng, Ke-qing Zhao

**Affiliations:** ^1^Key Laboratory of Medical Molecular Virology of Ministries of Education and Health, Department of Medical Microbiology and Parasitology, School of Basic Medical Sciences, Fudan University, Shanghai, China; ^2^Department of Otorhinolaryngology-Head and Neck Surgery, Eye and ENT Hospital, Shanghai Key Clinical Disciplines of otorhinolaryngology, Fudan University, Shanghai, China; ^3^Key Laboratory of Medical Molecular Virology, Huashan Hospital, Shanghai Medical College of Fudan University, Shanghai, China; ^4^Department of Laboratory Medicine, Hangzhou Medical College, Hangzhou, China; ^5^School of Preclinical Medicine, Wannan Medical College, Wuhu, China; ^6^Henan Engineering Lab of Antibody Medicine, Key Laboratory of Cellular and Molecular Immunology, Medical College of Henan University, Kaifeng, China; ^7^Department of Microbial Genetics, Faculty of Science, Interfaculty Institute of Microbiology and Infection Medicine Tübingen, University of Tübingen, Tübingen, Germany

**Keywords:** *Staphylococcus epidermidis*, biofilm, nicotine, tobacco smoke, two-component signal transduction system

## Abstract

*Staphylococcus epidermidis* is a common bacterial colonizer of human skin and mucous membranes, yet it has emerged as an important nosocomial pathogen largely due to its ability to form biofilms. Tobacco smoke has been demonstrated as a contributor to various infection diseases by improving the biofilm formation of multiple bacterial species; however, the association between tobacco smoke and *S. epidermidis* biofilm is still unclear. In this study, we tested the effect of nicotine, one of the most active components of tobacco, on *S. epidermidis* biofilm formation, and we studied the underlying mechanisms. Our results showed that nicotine promoted the biofilm formation of *S. epidermidis* 1457 strain (SE1457) and enhanced its initial attachment to a polyethylene surface as well as polysaccharide intercellular adhesin (PIA) production. In addition, an increased extracellular DNA release and a higher autolysis rate of SE1457 was detected after nicotine treatment, which was consistent with the increased ratio of dead cells in nicotine-treated SE1457 biofilm observed with confocal laser-scanning microscopy. Furthermore, the effect of nicotine on several autolysis-related and biofilm-related gene knockout mutants of SE1457 was tested. It showed that in *ΔsaeRS, ΔlytSR*, and *ΔsceD*, nicotine induced increase in biofilm formation was similar to that in SE1457; but in *ΔarlRS, ΔatlE*, and *ΔicaC*, the effect was obviously impaired. Consistently, the increase of the bacterial autolysis rate in *ΔarlRS* and *ΔatlE* induced by nicotine was not as significant as that in SE1457. Meanwhile, the growth inhibition of nicotine on SE1457 was observed, and it was much less on *ΔarlRS* and restored by the *arlRS* complementation. The *arlRS* transcription in SE1457 was inhibited by nicotine during cultivation as indicated by a promoter reporter assay using green fluoresent protein. Taken together, our study indicates that nicotine improves *S. epidermidis* biofilm formation by promoting its initial attachment and intercellular accumulation; the *arlRS, atlE*, and *ica* genes mediating bacterial autolysis and PIA production play an important role in this process.

## Introduction

*Staphylococcus epidermidis (S. epidermidis)* is an oppotunistic pathogen that commonly colonizes on the human skin and mocosal surfaces. Although *S. epidermidis* is usually considered part of the commensal flora, it has emerged as an important etiologic agent of nosocomial infections partially due to the increasing use of indwelling medical devices and immunosupressive drugs (Gotz, 2002; Ziebuhr et al., 2006).

The pathogenisis of *S. epidermidis* is associated with its ability to form biofilm, which is a multilayered structure containing microorganism communities that are attached to biotic or abiotic surfaces (Wu et al., 2012). Bacteria within biofilm are encased in self-produced matrix composed of extracellular polysaccharide, DNA, and proteins (Rabin et al., 2015). Given their high degree of resistance to the human immune system and current antimicrobial agents, bacterial biofilm plays an important role in the persistence of many chronic human infections (Parsek and Singh, 2003).

Bacterial biofilm formation can be influenced by diverse microenvironmental conditions (Van Wamel et al., 2007; Alves et al., 2010; Wang et al., 2015; Totani et al., 2017). Tobaco smoke, a contributor to various infectious diseases, has been shown to enhance bacterial biofilm formation in multiple species, such as *Staphylococcus aureus, Streptococcus pneumoniae*, and *Streptococcus mutans* (Hutcherson et al., 2015). Furthermore, several studies have demonstrated a positive relationship between nicotine, the major addictive component of tobacco smoke, and bacterial biofilm formation. For example, Huang et al. reported that nicotine could stimulate *Streptococcus mutans* biofilm formation and its metabolic activity (Huang et al., 2012; Li et al., 2014; Huang et al., 2015). They also found that nicotine could enhance *Streptococcus gordonii* biofilm formation, aggregation, and gene expression of binding proteins (Huang et al., 2014). However, the effect of nicotine on *S. epidermidis* and gene regulation remains unclear.

Bacterial two-component signal transduction systems (TCSs) serve as basic stimulus-sensing and response mechanisms by which bacteria adapt to environmental stresses and consequently play a vital role in pathogenesis, including biofilm formation (Groisman, 2016; Zschiedrich et al., 2016; Tiwari et al., 2017). In *S. epidermidis*, the TCSs ArlRS, Agr, LytSR, SaeRS, SrrAB, and YycFG (Zhu et al., 2010; Lou et al., 2011; Dai et al., 2012; Wu et al., 2012, 2014, 2015; Xu et al., 2017) have been reported to be involved in regulating biofilm formation. However, whether these TCSs are involved in regulating bacterial responses to nicotine remains unknown.

In this study, we tested the effect of nicotine on *S. epidermidis* biofilm formation. In addition, the underlying mechanism of nicotine-induced *S. epidermidis* biofilm was studied and the TCSs involved in this process were further tested, which may improve our knowledge on the relationship between the host microenvironment and *S. epidermidis* biofilm infection, and may help us to explore new therapeutic strategies.

## Materials and Methods

### Bacterial Strains, Plasmids, Primers and Culture Media

The bacterial strains used in this study are listed in Table 1. *S. epidermidis* 1457 (SE1457, Genome Accession Number: NZ_CP020463.1) was kindly provided by Dr. Yicun Gao from Hong Kong University. The gene knockout mutants were constructed in previous studies as well as in this work (Table 1). The *S. epidermidis* clinical strains were isolated from patients with chronic rhinosinusitis (CRS) in the Department of Otorhinolaryngology-Head and Neck Surgery, Eye and ENT Hospital of Fudan University. All of the strains were cultured in tryptone soy broth (TSB; OXOID, Basingstoke, United Kingdom). For the detection of the *S. epidermidis* biofilm formation, the strains were cultured in TSB medium supplemented with 0.5% glucose. For the recovery of staphylococcal cells after electroporation, B2 medium (1% casein hydrolysate, 2.5% yeast extract, 0.5% glucose, 2.5% NaCl, 0.1% K_2_HPO4, pH 7.5) was used. The vectors pMAD and pCM29 were used for the construction of gene knockout mutants and promoter-green fluoresent protein (GFP) reporter plasmid. When appropriate, antibiotics were used at the following concentrations: chloramphenicol (10 μg/ml), ampicillin (100 μg/ml), spectinomycin (100 μg/ml), and erythromycin (50 μg/ml). All the primers used in this study are listed in Table 2.

**Table 1:** Bacterial strains and plasmids used in this study.

Plasmids or strains	Description^a^	Source or reference	
**Plasmids**		
pMAD	A temperature-sensitive shuttle vector, (Amp^R^, Em^R^)	Arnaud et al., 2004	
pMAD-*ΔsceD*	Recombinant plasmid	This study	
pCM29	A shuttle vector (Amp^R^, Cm^R^)	Pang et al., 2010	
pCM-*arl-*P	The *arlRS* promoter region cloned into pCM29	This study	
**Bacterial strains**		
SE1457	A biofilm positive *S. epidermidis* clinical isolate, used as a wild-type strain	Mack et al., 1992	
*ΔarlRS*	An *arlRS* knockout mutant of SE1457	Wu et al., 2012	
ParlRS	*ΔarlRS* complemented with plasmid pCN51- *arlRS*	Wu et al., 2012	
*ΔatlE*	An *atlE* knockout mutant of SE1457	Qin et al., 2007	
*ΔicaC*	An icaC knockout mutant of SE1457	Wu et al., 2014	
*ΔlytSR*	A lytSR knockout mutant of SE1457	Zhu et al., 2010	
*ΔsaeRS*	A *saeRS* knockout mutant of SE1457	Lou et al., 2011	
*ΔsceD*	A *sceD* knockout mutant of SE1457	This study	
*E. coli* DC10B	mcrA Δ(mrr-hsdRMS-mcrBC) φ80lacZΔM15 ΔlacX74 recA1 araD139 Δ(ara-leu)7697 galU galK rpsL endA1 nupG Δdcm	Monk et al., 2012	

*^*a*^Amp^*R*^, ampicillin resistance; Cm^*R*^, chloramphenicol resistance; and Em^*R*^, erythromycin resistance*.

**Table 2:** Primers used in this study.

Primers	Sequence (5′→3′)	Restriction enzymes
**Primers for construction and verification of the *sceD* deletion mutant**		
sceD-US-F	GAAGATCTGACCAGTGAA CTAAGCTCAG	BglII
sceD -US-R	TCCCCCGGGTATTAAAAAT CCTCCTAAAAGTGAT	SmaI
sceD -DS-F	CGGAATTCTTAAAATATGAA GTATCTACCATCTTCTC	EcoRI
sceD -DS-R	CGGGATCCTCCCATTTTTC ATTAATATATGCCA	BamHI
Spc-F	TGGTTCAGCAGTAAATGGTGG	
Spc-R	CATCTGTGGTATGGCGGGTA	
sceD-In-F	GCTATAGGACTAGGCGTTG	
sceD-In-R	GCAGTTACCCAATGACCTGG	
**Primers for construction of *arlRS* promoter-GFP reporter plasmid**		
1457-arl-P-F	CACGCTAGCGACCAATCCAA AAGAACAACTTG	NheI
1457-arl-P-R	CTTGGTACCTACATCTTAAAC AATTAACTCGTATTTC	KpnI

### Biofilm Assay

The biofilm formation of *S. epidermidis* was detected by a microtiter plate assay as described elsewhere (Zhu et al., 2017). Briefly, bacterial strains were incubated overnight at 37°C to get to the stationary phase. The cultures were then diluted (1:200), and 200 μL of bacterial suspension was added into each well in a 96-well plate (Corning Inc., United States). To detect the effect of DNase I on biofilm formation, 5 μl DNase I (5U/μl, Takara Bio Inc., Japan) was added to the wells. The bacterial culture was incubated at 37°C for 24 h, then washed with PBS 3 times to remove all of the non-adherent cells. Afterward, 200 μL of methanol was added to each well to fix the attached bacteria at room temperature for 20 min, and then it was removed. Each well was air-dried, then filled with 200 μl of 2% crystal violet and incubated at room temperature for 10 min. After removing the excess dye, the wells were rinsed with running tap water until the water was colorless, and then remaining biofilms were incubated with 200 μL of 10% acetic acid with shaking for 1 h at room temperature. Finally, the optical density of each well was measured at 570 nm using a microtiter plate reader (DTX-880 Multimode Detector, Beckman Coulter, United States).

### Initial Bacterial Attachment Assay

Mid-exponential phase cells were washed with PBS three times, resuspened in TSB and TSB supplemented with nicotine, adjusted to OD_600_ = 0.1, and then incubated in the wells (1 ml per well) of a six-well polyethylene plate (Nunc, Thermo Fisher Scientific, United States) for 1 h at 37°C. The wells were washed with PBS 3 times gently and then the attached cells were photographed under a light microscope and counted by Image J software (National Institutes of Health, United States). For each sample, a minimum of six representative optical fields were randomly selected and counted.

### Observation of *S. epidermidis* Biofilms and PIA by CLSM

The effect of nicotine on the *S. epidermidis* biofilms was evaluated by LIVE/DEAD staining. Briefly, the biofilms were washed with PBS 3 times, and then stained with 1 μM of SYTO9, 1 μM of propidium iodide (PI), and 2.5 μg/ml Wheat Germ Agglutinin(WGA)-Alexa Fluor^®^ 350 conjugate (Thermo Fisher Scientific, United States) for 20 min. The stained cells and polysaccharide intercellular adhesin (PIA) were visualized by confocal laser-scanning microscopy (CLSM) (Leica TCS SP8 Confocal Laser Scanning Platform, Leica Microsystems, Germany) with a 63x 1.4-NA oil immersion objective. Three-dimensional biofilm images were created with IMARIS 7.0 software (Bitplane, United States). The red, green, and blue fluorescence intensity in each image was determined using Leica Application Suite 1.0 software (Leica Microsystem, Germany).

### Construction of Gene Knockout Mutants of *S. epidermidis*

The *sceD* deletion mutant was constructed by allelic replacement using the temperature-sensitive plasmid pMAD as described previously (Arnaud et al., 2004). In brief, the spectromycin-resistance cassette (*spc*, about 1 kb) digested with SmaI and BamHI endonucleases (Fermentas, Thermo Fisher Scientific, United States) was inserted into the pMAD plasmid, named as pMAD-*spc*. About 0.9-kb PCR products of flanking region of *sceD* were cloned into pMAD-*spc*. The recombinant plasmid was transferred into *Escherichia coli* DC10, and then into SE1457, followed by the process of allelic replacement and screening as performed previously (Wu et al., 2015). The *sceD* deletion mutant (*ΔsceD*) was verified by PCR and DNA sequencing.

### Determination of Bacterial Growth Curves

The growth curves of the *S. epidermidis* strains were determined by measuring the optical density at a wavelength of 600 nm using an automated growth curve detector (Biocreen C, Finland). Briefly, overnight cultures were diluted (1:200) and incubated at 37°C with shaking at 220 rpm. The OD_600_ of the bacterial culture was measured at 30 min intervals for 8.5 h.

### Detection of Triton X-100-Induced Bacterial Autolysis

A Triton X-100-induced autolysis assay was performed to determine the effect of nicotine on *S. epidermidis* autolysis. The overnight culture of SE1457 was diluted in fresh TSB containing 1 M NaCl, and the bacteria were grown to the mid-exponential phase (OD_600_ = 0.6–0.8), washed twice in cold sterile distilled water, resuspended in the same volume of 0.05 M Tris-HCl containing 0.05% Triton X-100 (pH 7.2), and incubated at 30°C. The optical density at 600 nm was measured every 30 min. The Triton X-100-induced autolysis rate was calculated as follows: Ra = OD_0_-OD_t_/OD_0_.

### Quantification of Extracellular DNA

Extracellular DNA (eDNA) in *S. epidermidis* was quantified using the method previously described with modifications (Allesen-Holm et al., 2006; Qin et al., 2007). In brief, overnight cultures of *S. epidermidis* strains were diluted to OD_600_ = 0.001 in the minimal growth medium supplemented with 0.5% glucose, 10% TSB, and 0.05 mM PI. The diluted cultures were transferred to the wells of a polystyrene microtiter plate (200 μL/well) and incubated at 37°C for 24 h. The cell density was measured at OD_600_ using a microtiter plate reader (BioRAD, United States). The fluorescence of PI-bound eDNA was measured using a Varioskan^TM^ LUX multimode microplate reader (Thermo Fisher, United States) with the excitation/emission wavelength at 535/610 nm. Relative amounts of eDNA per OD_600_ unit were calculated.

### Detection of *arlRS* Expression Using a Promotor-GFP Reporter Plasmid

The *S. epidermidis arlRS* promotor-GFP transcriptional reporter was constructed using the shuttle vector pCM29 (Pang et al., 2010). A ∼200-bp fragment containing the putative *arlRS* promoter was amplified from SE1457 genomic DNA using primers 1457-arl-P-F and 1457-arl-P-R. The PCR products were digested with NheI and KpnI, and subsequently ligated upstream of the GFP gene in pCM29 to generate the plasmid pCM-*arl*-P, which was transformed into DC10B and then SE1457 by electroporation. To monitor the *arlRS* expression, SE1457 containing pCM-*arl*-P was cultivated in TSB to OD_600_ = 0.8, and then incubated with or without 4000 μg/ml nicotine at 37°C with shaking, respectively. Bacterial cultures were collected at different time points, and centrifuged. The pellets were washed three times with normal saline and resuspended in normal saline with OD_600_ = 0.6. The bacterial suspension was transferred to a black 96-well plate and the fluorescence intensity was measured using a Victor X5 multilabel plate reader (PerkinElmer, Inc., United States) with excitation at 480 nm and emission at 515 nm. Values from quadruplicate wells were averaged, and the experiment was repeated at least once.

### Data Analysis

Data are expressed as mean ± standard deviation. Unpaired two-tailed *t*-test was used for between-group analyses. One-way ANOVA followed by Boneferroni’s *post hoc* tests were exploited for the comparison of three or more groups. All of the analyses were performed using GraphPad Prism 7 (GraphPad Software, Inc., La Jolla, CA, United States). Two-tailed *P*-values < 0.05 were considered to be statistically significant.

## Results

### Nicotine Improves *S. epidermidis* Biofilm Formation

**FIGURE 1:**
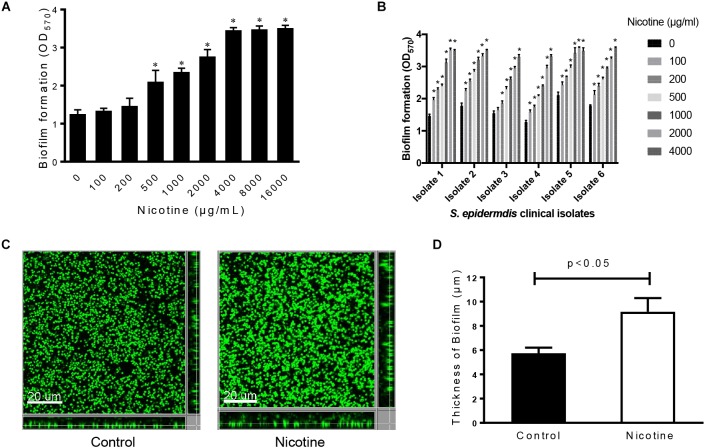
Effect of nicotine on biofilm formation of *S. epidermidis* strains. **(A)** SE1457 strain was exposed to various concentration of nicotine (0, 100, 200, 500, 1000, 2000, 4000, 8000, 16000 μg/ml). The biofilm formation of *S. epidermidis* was detected by a microtiter plate assay by measuring crystal violet stained biofilm at OD_570_. **(B)** Six biofilm-forming *S. epidermidis* clinical isolates were exposed to different concentration of nicotine (0, 100, 200, 500, 1000, 2000, 4000 μg/ml), and the biofilm formation was detected by a microtiter plate assay. **(C)** SE1457 biofilms without nicotine treatment (Control) or with 4000 μg/ml nicotine treatment (Nicotine) were staining with SYTO9 and observed by confocal laser scanning microscopy (CLSM). **(D)** The thickness of biofilms in no nicotine treatment and 4000 μg/ml nicotine treatment groups was analyzed using Leica Application Suite 1.0 software. The experiment was performed in triplicates (^∗^indicates statistically significant, *P* < 0.05).

In order to determine the effects of nicotine on *S. epidermidis* biofilm formation, SE1457 strain was exposed to different concentrations of nicotine (0, 100, 200, 500, 1000, 2000, 4000, 8000, and 16000 μg/ml) for 24 h at 37°C. A dose-dependent nicotine-induced increase in the biofilm formation was observed. The maximal biofilm formation was obtained in the 4000–16000 μg/ml nicotine groups (Figure 1A). The effect of nicotine on biofilm formation was further tested in six clinical isolates of *S. epidermidis*. Similar to the effect on SE1457, 500–4000 μg/ml of nicotine dramatically enhanced biofilm growth in all six clinical isolates. In 100 μg/ml of nicotine treatment group, biofilm formation in five isolates showed a significant increase (*P* < 0.05), compared to the untreated control (Figure 1B).

To explore mechanisms of nicotine induced biofilm enhancement, we chose 4000 μg/ml as the working concentration of nicotine in the following experiments, which is consistent with previous studies (Huang et al., 2012, 2015).

To confirm the positive effect of nicotine on SE1457 biofilm formation, the thickness of the mature biofilms was measured. The representative image is shown in Figure 1C; dense biofilm was formed in the nicotine-treated group (9.17 ± 1.96 μm, *n* = 3) compared to the control (5.75 ± 0.79 μm) (*p* < 0.05) (Figure 1D).

### Nicotine Increases the Initial Attachment and PIA Production of *S. epidermidis*

**FIGURE 2:**
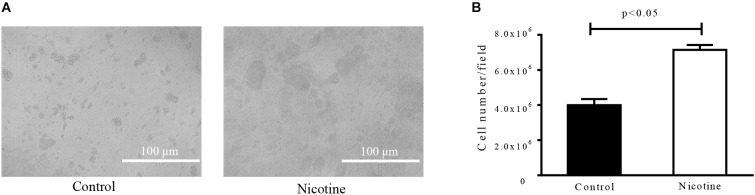
Effect of nicotine on initial attachment of *S. epidermidis*. Primary attachment of the SE1457 to polystyrene surfaces. Overnight cultures grown to an OD600 of 0.6 were diluted with TSB (Control) and TSB supplemented with 4000 μg/ml nicotine (Nicotine) to an OD_600_ of 0.1 in PBS and inoculated into a six-well polyethylene plate for 1 h at 37°C. The attached cells were photographed under a light microscope **(A)** and the cell numbers were counted by Image J software **(B)**.

Since the biofilm formation process is composed of two major steps, the initial attachment and the following bacterial accumulation, the impact of nicotine on both steps was investigated. To test the effect of nicotine on bacterial primary attachment, the cells attached to the polysterene wells with or without nicotine treatment were pictured and counted (Figure 2A). More cells of SE1457 attached to the bottom of the polyethylene wells after 4000 μg/ml nicotine treatment (7.15 ± 0.56 × 10^6^, *n* = 4) than the untreated control (4.00 ± 0.70 × 10^6^, *n* = 4). This difference was statistically different (*p* < 0.05) (Figure 2B).

**FIGURE 3:**
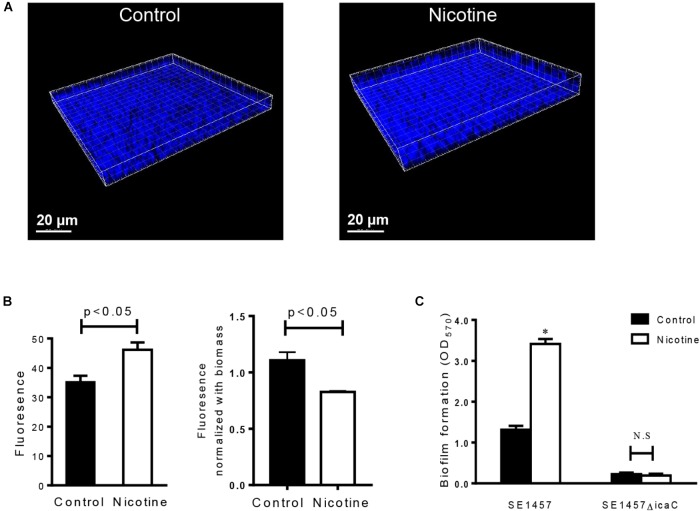
Effect of nicotine on PIA production and biofilm formation of the *icaC* mutant. **(A)** PIA in SE1457 biofilms without nicotine treatment (Control) or with 4000 μg/ml nicotine treatment (Nicotine) was stained with Alexa Fluor^®^ 350 conjugate of wheat germ agglutinin and observed by CLSM (shown in blue fluorescence). **(B)** Left: The fluorescence intensity was analyzed using Leica Application Suite 1.0 software; right: PIA fluorescence was normalized by the total biofilm biomass. **(C)** Effect of nicotine on biofilm formation of the *icaC* knockout mutant of SE1457 was detected by a microtiter plate assay; ^∗^*P* < 0.05; N.S, no significance.

To test the effect of nicotine on bacterial intercellular adhesion, the production of PIA, the major component that is synthesized through the cooperation of the proteins that are encoded by the ica operon (Bartoszewicz et al., 2004), was detected using Alexa Fluor^®^ 350 conjugate of WGA. It showed that the amount of PIA after nicotine treatment was increased *in situ* (Figure 3A), and the difference between the two groups was significant [the nicotine treatment group was 46.17 ± 4.30 and the control group was 35.26 ± 3.53 (*n* = 3) (*p* < 0.05)] (Figure 3B). However, ratio of the PIA fluorescence normalized by the total biofilm biomass was slightly lower in the nicotine treatment group (Figure 3B). The enhancement of nicotine-induced biofilm formation was inhibited by knocking out the *icaC* gene (Figure 3C, *ΔicaC*), compared to the wild-type (WT) strain.

### Nicotine Increases the Release of eDNA by *S. epidermidis*

**FIGURE 4:**
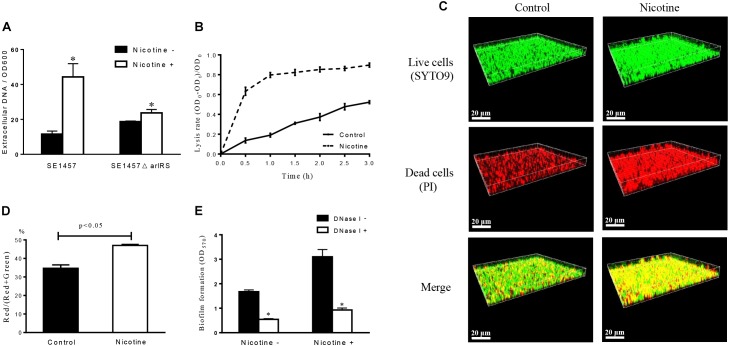
Effect of nicotine on extracellular DNA release and bacterial autolysis. **(A)** The amount of eDNA in SE1457 WT and Δ*arlRS* strains biofilms with and without 4000 μg/ml nicotine treatment was determined by measuring the fluorescence of PI-bound eDNA with the excitation/emission wavelength at 535/610 nm. Relative amounts of eDNA per OD600 unit were calculated. The experiment was performed in triplicates. (^∗^*P* < 0.05) **(B)** Detection of Triton X-100-induced bacterial autolysis of SE1457 with or without nicotine treatment; OD_600_ readings were measured every 30 min. **(C)** SE1457 biofilms were grown in a FluoroDish for 24 h with or without nicotine, then stained with SYTO9 (green fluorescence) and PI (red fluorescence) to represent the live and dead bacteria independently. **(D)** The fluorescence intensity was analyzed using Leica Application Suite 1.0 software, and the ratio of Red/(Red + Green) was calculated. **(E)** The biofilm formation of *S. epidermidis* SE1457 strain was detected using a microtiter plate assay by measuring crystal violet stained biofilm at OD_570_. DNase I was added to the wells (25 U/well) in both 4000 μg/ml nicotine treatment group and the control at the time of bacterial inoculation.

After 24 h of treatment, the amount of eDNA in the *S. epidermidis* biofilms was measured by detecting the fluorescence of PI-bound eDNA at the wavelength of 610 nm. The eDNA concentrations were increased in the nicotine-treated group (44.33 ± 7.60, *n* = 3) compared to the control group (11.58 ± 1.74, *n* = 3) (*p* < 0.05) (Figure 4A).

### Nicotine Enhances the Autolysis Rate of *S. epidermidis*

To examine the effect of nicotine on *S. epidermidis* autolysis, the Triton X-100-induced autolysis curve was determined on SE1457. Following the addition of 0.05% Triton X-100, the cultures with the addition of 4000 μg/ml of nicotine exhibited a much higher autolysis rate than the control group, and the difference between the two groups was significant (Figure 4B), which indicated that nicotine could enhance the autolysis rate of SE1457. This was confirmed by determining the viability of SE1457 *in situ* with or without nicotine treatment in 24 h biofilms by CLSM with LIVE/DEAD staining (viable cells are stained by SYTO9 are shown in green and dead cells stained by PI are displayed in red). The representative staining of biofilms with or without nicotine treatment is shown in Figure 4C. The red and green fluorescence intensities were quantified by stack profile in different channels, respectively. The number of red/(red + green) in the nicotine-treated group and the untreated control group were 47.00 ± 1.00% (*n* = 3) and 34.67 ± 3.22% (*n* = 3) (*p* < 0.05) independently, which indicated that there was a higher ratio of dead cells in the nicotine-treated group compared to the control group (Figure 4D).

### DNase I Inhibits Nicotine Induced Biofilm Formation

To elucidate if the enhanced biofilm formation of *S. epidermidis* after nicotine treatment is associated with the increased eDNA release, we detected the effect of DNase I on biofilm formation in the 1457 WT strain with and without 4000 μg/ml nicotine treatment. It showed that the nicotine induced biofilm formation was significantly inhibited by DNase I (25U/well): in nicotine treatment group, the biofilm formation indicated by OD_570_ was decreased from 3.10 ± 0.30 to 0.93 ± 0.08 (*n* = 3, *p* < 0.05), while in nicotine untreated group OD_570_ was decreased from 1.68 ± 0.07 to 0.544 ± 0.03 (*n* = 3, *p* < 0.05) (Figure 4E).

### Knocking Out *arlRS* or *atlE* Inhibits Nicotine-Induced Biofilm Formation

**FIGURE 5:**
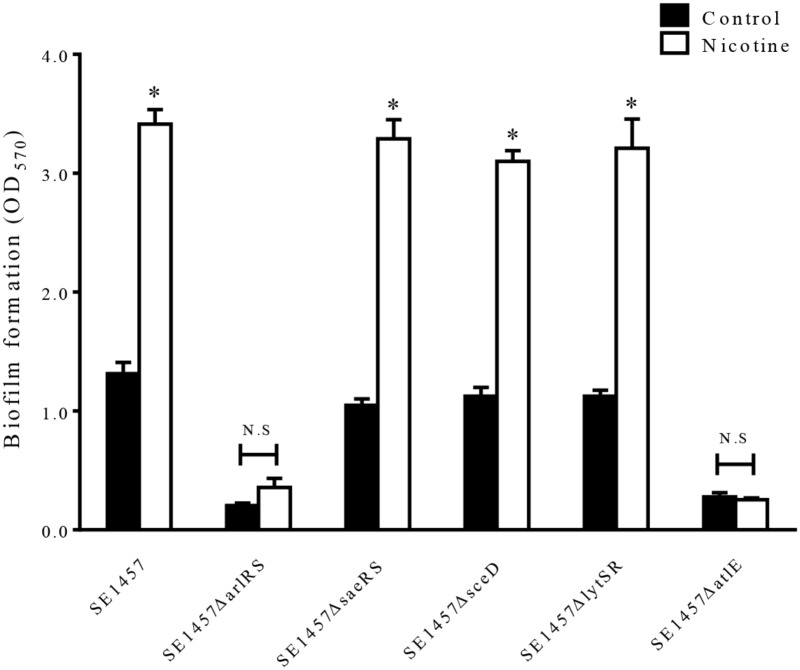
Effect of nicotine on biofilm formation of *S. epiderimidis* 1457 wild-type strain and biofilm-related gene knockout mutants. The 24 h biofilms of the *ΔarlRS, ΔsaeRS, ΔsceD, ΔlytSR*, and *ΔatlE* mutants of SE1457 with or without nicotine treatment were detected by a microtiter plate assay and compared to that of the parent strain. ^∗^*P* < 0.05 compared with their own controls in each strain; N.S, no significance.

To elucidate the molecular basis of the increased autolysis by nicotine, we constructed the gene knockout mutants *ΔarlRS, ΔlytSR, ΔsaeRS, ΔatlE* (Qin et al., 2007) and *ΔsceD*, using SE1457 as the parent strain, and then compared the biofilm formation with or without nicotine treatment in these strains. This showed that nicotine-induced biofilm formation was inhibited in the *ΔarlRS* and *ΔatlE* groups while no obvious differences were found among the WT strain and the other mutants (Figure 5).

### Knocking Out *arlRS* or *atlE* Affects Nicotine-Induced Bacterial Autolysis

**FIGURE 6:**
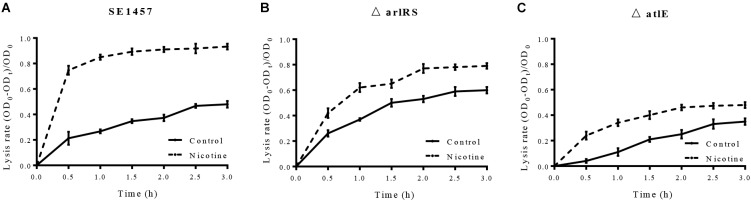
**(A–C)** Effect of nicotine on Triton X-100-induced autolysis of the SE1457, *ΔarlRS* and *ΔatlE* strains. *S. epidermidis* strains SE1457, *ΔarlRS* and *ΔatlE* were incubated with 0.1% Triton X-100 for 3 h without nicotine (Control) or with 4000 μg/ml nicotine (Nicotine). The lysis rate was calculated at 30 min intervals and the increase of the lysis rate was determined by calculating the difference of the values of the lysis rate between the nicotine group and the control group at the 3 h point.

To investigate the effect of mutations of *arlRS* and *atlE* on nicotine-induced bacterial autolysis, WT SE1457, *ΔarlRS*, and *ΔatlE* strains were incubated with 0.1% Triton X-100 for 3 h with or without nicotine. The lysis rate was calculated at 30 min intervals and the increase of the lysis rate was determined by calculating the difference of the values of the lysis rate between the nicotine group and the control group at the 3 h point. Our results showed that the increase of the lysis rate was impaired in the *ΔarlRS* and *ΔatlE* groups compared to that in the WT group (Figure 6).

### Knocking Out *arlRS* Impairs Growth Inhibition and eDNA Release Induced by Nicotine

**FIGURE 7:**
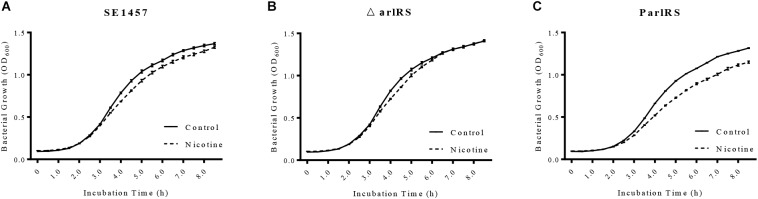
Effect of nicotine on bacterial growth of SE1457, *arlRS* knockout mutant and complementation strains. **(A–C)** The growth curves of the *S. epidermidis* WT strain SE1457, the *arlRS* knockout mutant (*ΔarlRS)* and the *arlRS* complementation strain (ParlRS) in TSB without nicotine (Control) or with 4000 μg/ml nicotine (Nicotine) were determined by measuring the OD_600_ of the bacterial culture for 8.5 h at 30 min intervals.

Since nicotine increased the autolysis of *S. epidermidis*, its impact on bacterial growth was further investigated by detecting the growth curves of SE1457 in TSB and TSB supplemented with nicotine. SE1457 growth in the TSB with nicotine was slower than that in TSB, especially in the exponential phase (Figure 7A), indicating a growth inhibition effect of nicotine. The inhibition on growth by nicotine was impaired and restored in the *arlRS* knockout and complementation strains, respectively (Figures 7B,C). We further tested the eDNA concentrations in the *arlRS* mutant with and without nicotine treatment. After nicotine treatment, eDNA concentration normalized with OD_600_ was increased from 18.71 ± 0.39 to 23.73 ± 1.97 in *arlRS* mutant (Figure 4A).

### Nicotine Inhibits *arlRS* Expression in *S. epidermidis*

**FIGURE 8:**
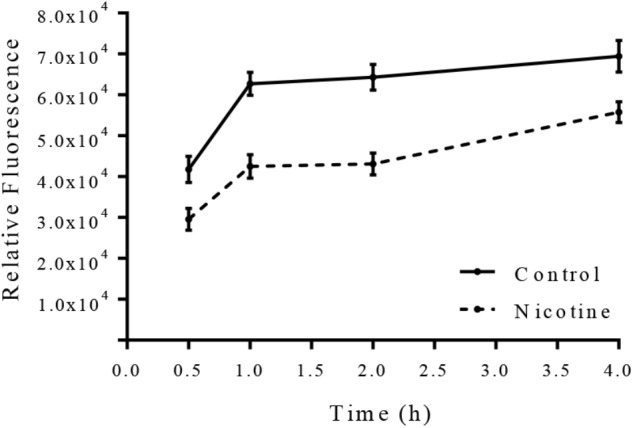
Effect of nicotine on *arlRS* expression in SE1457. SE1457 containing an *arlRS* promotor-GFP reporter plasmid (pCM-*arl*-P) was incubated with or without 4000 μg/ml nicotine at 37°C with shaking, and bacterial cultures were collected at different time points to detect the fluorescence intensity with excitation at 480 nm and emission at 515 nm. Values from quadruplicate wells were represented as mean ± standard deviation.

To detect the impact of nicotine on the *arlRS* expression in *S. epidermidis*, an *arlRS* promoter-GFP reporter system was constructed in SE1457, and its GFP fluorescence intensity was monitored after nicotine treatment. As shown in Figure 8, after adding 4000 μg/ml nicotine to the SE1457 culture, the expression of *arlRS* indicated by fluorescence intensity was decreased immediately and remained at a low level for at least 4 h compared to the non-treatment control, suggesting that the function of *arlRS* was inhibited by nicotine.

## Discussion

The ability to form biofilms on indwelling devices and human mucosal surfaces is an important pathogenic factor of *S. epidermidis*, which is the major microbe in catheter-related infections (Mack et al., 2006). Recently, the pathogenic effect of *S. epidermidis* biofilm in chronic rhinosinusitis (CRS) has received increasing attention due to the frequent isolation of the bacterium in CRS patients (Sachse et al., 2008; Pandak et al., 2011). In this study, we demonstrated that nicotine enhances *S. epidermidis* biofilm formation *in vitro*, and this impact involves multiple genes that are associated with bacterial autolysis and biofilm formation, including *arlRS, atlE*, and *ica* operons.

Nicotine, an alkaloid, is one of the most toxic chemical in tobacco (Huang et al., 2012). During smoking, nicotine can be easily absorbed through nasal mucosa (Temple, 1976) and is able to cross the biological membranes then get into the circulatory system and tissues. It has been detected in different human body systems, such as saliva and blood (Dhar, 2004; Yildiz, 2004). The presence of nicotine in nasal mucus, tissues and blood may be a risk factor for CRS patients and patients with implanted devices who are potentially exposed to normal flora, including *S. epidermidis.* As an evidence, tobacco smoke has been reported to correlate with CRS and the poor sinus surgery outcome (Lieu and Feinstein, 2000; Ebbert et al., 2007). In this study, we detected the effect of nicotine on SE1457 and the clinical *S. epidermidis* strains from CRS patients. In the nicotine-treated group of our study, a concentration-dependent upregulation of *S. epidermidis* biofilm formation *in vitro* was observed using a microtiter plate assay, and it was confirmed by CLSM observation that *S. epidermidis* formed a thicker biofilm when nicotine was present in the culture medium. We also found that stimulation of *S. epidermidis* biofilm formation by nicotine was more obvious when the polyethylene plates were coated with human fibrinogen or when nicotine treatment was extended from 24 to 48 h, even if lower concentrations of nicotine were used (Supplementary Figures S1, S2). These results indicate that the effect of nicotine on *S. epidermidis* biofilm formation may be more significant *in vivo*, especially in the condition of long time exposure to nicotine.

The development of biofilm formation is a two-step process involving primary attachment and intercellular adhesion phase. Our results indicate that nicotine has a positive effect on both steps. First, our results showed that nicotine significantly increased the initial attachment of SE1457. Second, nicotine treated biofilms contained more PIA, which serves as one of the major component in mature *S. epidermidis* biofilms.

In the step of bacterial primary attachment to organic or inorganic surfaces, eDNA plays an important role as proven by the observation that the removal of eDNA with DNase I significantly reduces initial bacterial adhesion (Das et al., 2010). In this work, enhanced eDNA release of *S. epidermidis* 1457 strain was observed after nicotine treatment, compared to the untreated control. Furthermore, the nicotine induced biofilm formation was significantly inhibited by DNase I, suggesting that eDNA was associated with enhanced bacterial attachment and biofilm formation in the nicotine-treated group. This finding is consistent with Kulkarni’s study that DNase I could abolish cigarette smoke-induced biofilm in the *Staphylococcus aureus* strain (Kulkarni et al., 2012). Bacterial cells lysis is the major source of eDNA release (Qin et al., 2007). Thus, we further assumed that bacterial autolysis might be enhanced by nicotine. Our results showed that nicotine did increase the autolysis rate of *S. epidermidis*, and it was further confirmed by the CLSM observation that there was a high ratio of dead cells in the nicotine-treated biofilms.

In the intercellular adhesion phase of *S. epidermidis* biofilm development, multiple factors including PIA and Aap (accumulation-associated protein) are involved. PIA is vital for biofilm formation in high shear stress conditions (Schaeffer et al., 2016) and its biosynthesis, exportation and modification are mediated by the products of the *icaADBC* operon (Mack et al., 1996). Our previous study showed that the *icaC* deletion in SE1457 abolished its PIA production and biofilm formation (Wu et al., 2014). In this work, we found that nicotine-treated biofilm contained larger amount of PIA, however, ratio of the PIA fluorescence normalized by the total biofilm biomass was slightly lower. It can be explained by the decreased expression of *arlRS* after nicotine exposure, since ArlRS is a positive regulator of PIA synthesis (Wu et al., 2012). Furthermore, enhanced biofilm formation in SE1457 induced by nicotine was abolished in *ΔicaC*. These results indicate that the *ica*-dependent pathway is involved in nicotine-induced *S. epidermidis* biofilm formation. The effect of nicotine on the expression of factors in *ica*-independent pathways, especially Aap, which contributes to foreign body infections caused by *S. epidermidis* (Schaeffer et al., 2015), needs future investigation.

Altered bacterial autolysis, eDNA release, and PIA synthesis after nicotine treatment suggests that signal sensing and regulation systems may participate in this process (Gilpin et al., 1972; Tobin et al., 1994; Yabu and Kaneda, 1995; Wells and Russell, 1996). TCSs are vital for bacteria to adapt to diverse niches by sensing the environmental stimuli with a membrane-associated histidine kinase and modulating gene expression with a cytoplasmic response regulator (von Eiff et al., 2002). Previous studies by our group found that in *S. epidermidis*, LytSR, SaeRS, and ArlRS TCSs modulate bacterial autolysis, while ArlRS, SrrAB, and YycGF TCSs regulate PIA synthesis (Zhu et al., 2010; Lou et al., 2011; Dai et al., 2012; Wu et al., 2012, 2014, 2015; Xu et al., 2017).

In this work, we found that among these TCSs, ArlRS plays a key role in regulating nicotine-associated biofilm enhancement. In comparing the effects of nicotine on SE1457 and its isogenic *arlRS* knockout mutant strain *ΔarlRS*, we found that the deletion of *arlRS* significantly inhibited not only nicotine-induced biofilm formation but also nicotine-induced growth inhibition, both of which were restored by the *arlRS* complementation strain. In addition, the nicotine-induced upregulation of the autolysis rate and eDNA release were significantly impaired in *ΔarlRS* as compared to that in the WT strain. Furthermore, the presence of nicotine inhibited *arlRS* expression, which is consistent with the results that *ΔarlRS* showed an increased Triton X-100-induced autolysis rate compared to its parent control, SE1457. These results suggest that nicotine-induced *S. epidermidis* biofilm formation is at least partially regulated by ArlRS.

The impact of nicotine on *S. epidermidis* biofilm formation may involve other functional genes or regulators, including *atlE* and *ica* operons. AtlE is the major autolysin in *S. epidermidis* and it is responsible for most of the eDNA release in cultures and biofilms (Qin et al., 2007). In this work, the deletion of *atlE* dramatically inhibited the nicotine-induced enhancement of biofilm formation as well as the upregulation of the bacterial autolysis rate. The microarray data showed that the expression of *atlE* was altered in the *arlRS* mutant as compared to that in SE1457 (data not shown), indicating that *arlRS* may play a role in *atlE*-mediated eDNA release.

*Staphylococcus epidermidis* ArlRS shares a high sequence similarity with the orthologues in other bacteria including *Staphylococcus aureus* (ArlRS), *Streptococcus pneumoniae* (CsrRS) and *Mycobacterium abscessus* (MtrAB). *S. aureus* is present in human nasal cavity and skin, and *S. pneumoniae* is found normally in the upper respiratory tract including the throat and nasal passages. Biofilm forming ability of *S. aureus* and *S. pneumoniae* strains is considered to play an important role during their colonization and infection. Since nicotine has a relatively high concentration in nasal mucus and saliva, it may have an impact on the biofilm formation and pathogenesis of the two human pathogens. We have found that nicotine inhibits *arlRS* transcription in *S. aureus*, similar with the result in *S. epidermidis* (unpublished data).

## Conclusion

Our study showed that nicotine, an active component of tobacco smoke, enhances *S. epidermidis* biofilm formation *in vitro* and indicates that the *arlRS, atlE*, and *ica* operons play important roles in this process by altering the bacterial autolysis, eDNA release, and PIA production. However, whether other regulatory systems are involved in this process warrants further investigation.

## Author Contributions

YW, K-qZ, DQ, C-qZ, and FG designed the work and revised the manuscript. YW, YM, TX, Q-zZ, JB, JW, TZ, and QL completed all the experiments. K-qZ and YW performed the statistical analysis, made the figures, and wrote the manuscript.

## Conflict of Interest Statement

The authors declare that the research was conducted in the absence of any commercial or financial relationships that could be construed as a potential conflict of interest.

## Supplementary Material

FIGURE S1 Effect of nicotine on the 24 h biofilm formation of *S. epidermidis* 1457 strain in the fibrinogen-coated 96-well polyethylene plates. SE1457 strain was exposed to various concentration of nicotine (0, 0.025, 0.05, 0.5, 5, 50, 500 μg/ml) for 24 h. The biofilm formation of *S. epidermidis* was detected by a microtiter plate assay. Biofilms were stained with crystal violet, dissolved in 200 μL of 10% acetic acid. After 10-fold dilution, the solution was measured at OD_570_ (^∗^*P* < 0.05).

FIGURE S2 Effect of nicotine on the 48 h biofilm formation of *S. epidermidis* 1457 strain in the tissue-culture treated 96-well polyethylene plates. SE1457 strain was exposed to various concentration of nicotine (0, 0.025, 0.05, 0.5, 5, 50, 500 μg/ml) for 48 h. Biofilms were stained with crystal violet, dissolved in 200 μL of 10% acetic acid. After 10-fold dilution, the solution was measured at OD_570_ (^∗^*P* < 0.05).
