# Supplementary figures and images for: Nicotine Enhances Staphylococcus epidermidis Biofilm Formation by Altering the Bacterial Autolysis, Extracellular DNA Releasing, and Polysaccharide Intercellular Adhesin Production
Source: Front Microbiol. 2018 Oct 29;9:2575. doi: 10.3389/fmicb.2018.02575 (PMC6215848; doi:10.3389/fmicb.2018.02575)

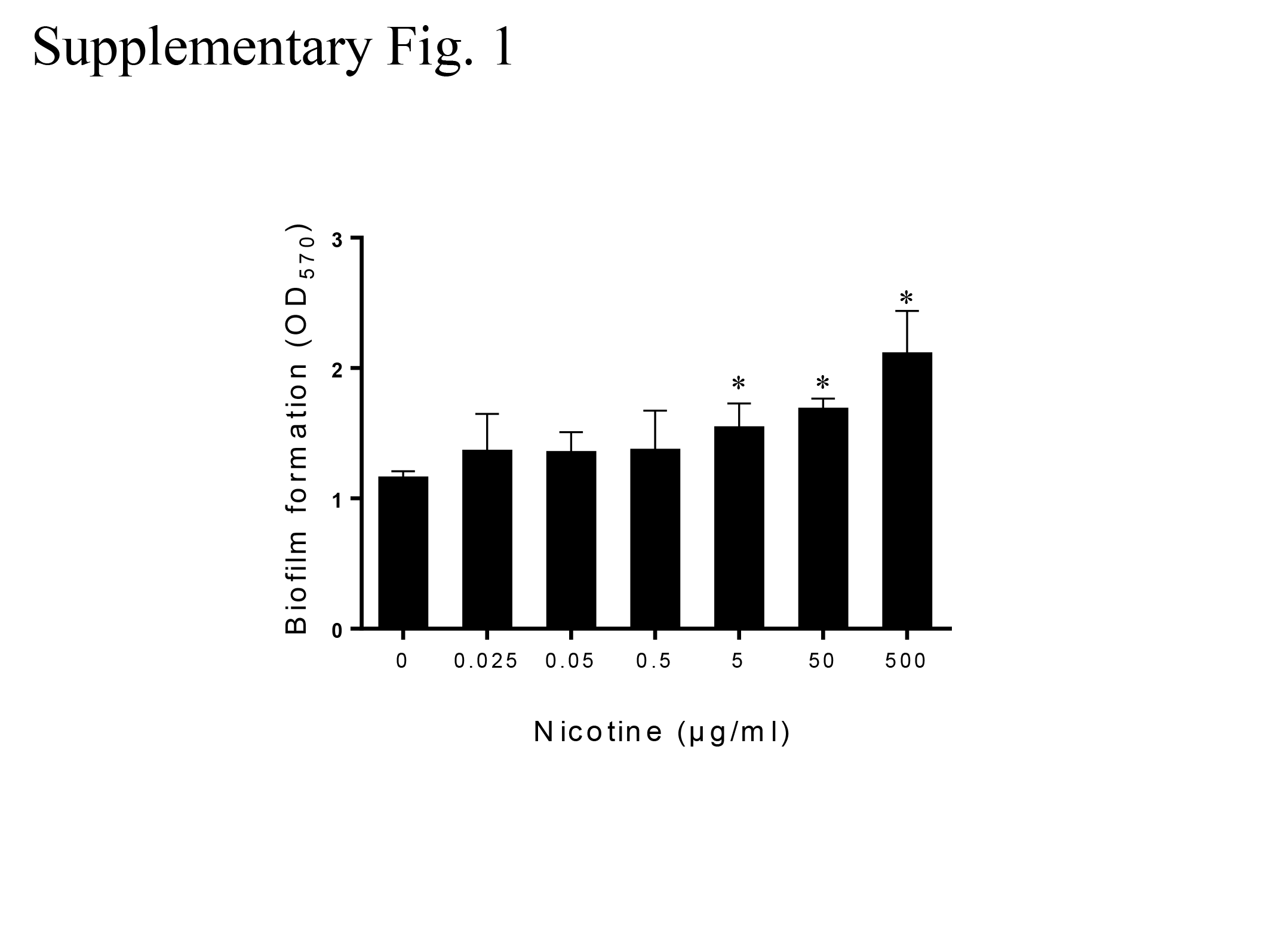

Supplement: FIGURE S1 — Effect of nicotine on the 24 h biofilm formation of S. epidermidis 1457 strain in the fibrinogen-coated 96-well polyethylene plates. SE1457 strain was exposed to various concentration of nicotine (0, 0.025, 0.05, 0.5, 5, 50, 500 μg/ml) for 24 h. The biofilm formation of S. epidermidis was detected by a microtiter plate assay. Biofilms were stained with crystal violet, dissolved in 200 μL of 10% acetic acid. After 10-fold dilution, the solution was measured at OD570 (∗P < 0.05). [file Image_1.TIF]

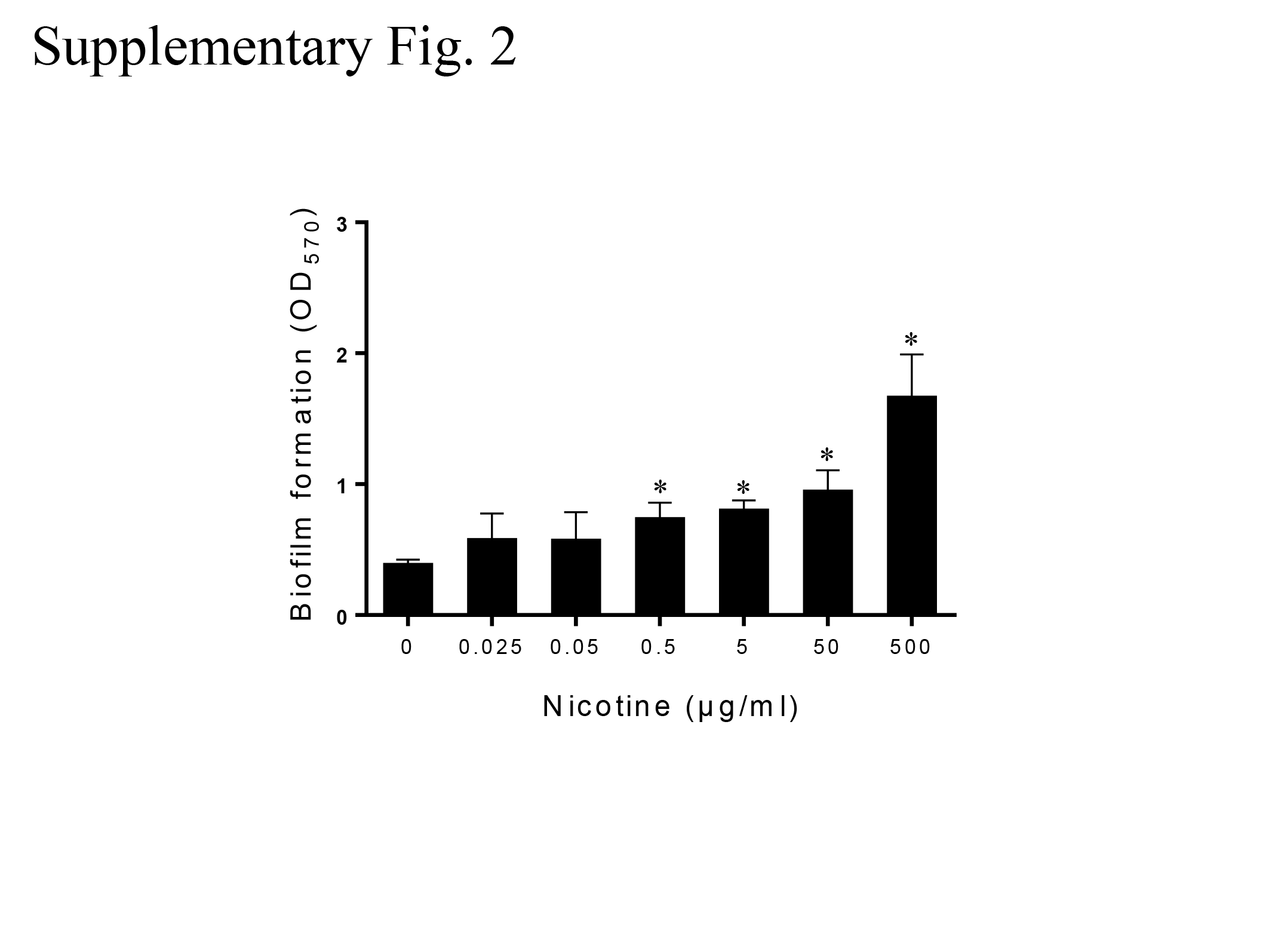

Supplement: FIGURE S2 — Effect of nicotine on the 48 h biofilm formation of S. epidermidis 1457 strain in the tissue-culture treated 96-well polyethylene plates. SE1457 strain was exposed to various concentration of nicotine (0, 0.025, 0.05, 0.5, 5, 50, 500 μg/ml) for 48 h. Biofilms were stained with crystal violet, dissolved in 200 μL of 10% acetic acid. After 10-fold dilution, the solution was measured at OD570 (∗P < 0.05). [file Image_2.TIF]
